# Influence of Exercise on Bone Remodeling-Related Hormones and Cytokines in Ovariectomized Rats: A Model of Postmenopausal Osteoporosis

**DOI:** 10.1371/journal.pone.0112845

**Published:** 2014-11-13

**Authors:** Lihui Li, Xi Chen, Shuang Lv, Miaomiao Dong, Li Zhang, Jiaheng Tu, Jie Yang, Lingli Zhang, Yinan Song, Leiting Xu, Jun Zou

**Affiliations:** 1 School of Kinesiology, Shanghai University of Sport, Shanghai, China; 2 Scientific Research Department, Shanghai University of Sport, Shanghai, China; 3 Medical School of Ningbo University, Ningbo, China; 4 School of Sports Science, Wenzhou Medical University, Wenzhou, China; Université de Lyon - Université Jean Monnet, France

## Abstract

This study aims to explore the effects of exercise on postmenopausal osteoporosis and the mechanisms by which exercise affects bone remodeling. Sixty-three Wistar female rats were randomly divided into five groups: (1) control group, (2) sham-operated group, (3) OVX (Ovariectomy) group, (4) DES-OVX (Diethylstilbestrol-OVX) group, and (5) Ex-OVX (Exercise-OVX) group. The rat osteoporosis model was established through ovariectomy. The Ex-OVX rats were made to run 251.2 meters every day, 6 d/wk for 3 months in a running wheel. Trabecular bone volume (TBV%), total resorption surface (TRS%), trabecular formation surface (TFS%), mineralization rate (MAR), bone cortex mineralization rate (mAR), and osteoid seam width (OSW) were determined by bone histomorphometry. The mRNA and protein levels of interleukin-1β (IL-1β_2_), interleukin-6 (IL-6), and cyclooxygenase-2 (Cox-2) were determined by *in situ* hybridization and immunohistochemistry, respectively. Serum levels of estrogen estradiol (E_2_), calcitonin (CT), osteocalcin (BGP), and parathyroid hormone (PTH) were determined by ELISA assays. The investigation revealed that compared to the control and the sham-operated groups, the OVX group showed significantly lower levels of TBV%, E_2_, and CT, but much higher levels of TRS%, TFS%, MAR, OSW, BGP, and PTH. The Ex-OVX group showed increased TBV% and serum levels of E_2_ and CT compared to the OVX group. Ovariectomy also led to a significant increase in IL-1β mRNA and protein levels in the bone marrow and IL-6 and Cox-2 protein levels in tibias. In addition, the Ex-OVX group showed lower levels of IL-1 mRNA and protein, IL-6 mRNA, and Cox-2 mRNA and protein than those in the OVX group. The upshot of the study suggests that exercise can significantly increase bone mass in postmenopausal osteoporosis rat models by inhibiting bone resorption and increasing bone formation, especially in trabecular bones.

## Background

Osteoporosis is a major health problem among the elderly and is characterized by low bone mineral density (T score for bone mineral density below 2.5) and microarchitectural deterioration of bone, leading to increased fracture risks [1]. Osteoporosis is prevalent among women, in particular postmenopausal women. Estrogen shortage/deficiency results in an increase in bone turnover, wherein the rate of osteoclastic resorption exceeds the rate of osteoblastic osteogenesis, leading to a net loss of bone mass. Many studies have indicated that hormone replacement therapies (HRT) can be used to prevent and/or treat postmenopausal osteoporosis. However, long-term estrogen supplementation poses several risks, e.g., endometrial cancer, breast cancer, ovarian cancer, and cardiovascular diseases, and is no longer recommended [2], [3].

Previous studies suggest that estrogen may inhibit osteoclast activity through regulating the levels of serum CT and PTH [4]. Estrogen stimulates the synthesis of CT, which directly inhibits osteoclast differentiation and activity, and thus reduces the number of osteoclasts and serum concentration of Ca^2+^ [5]. PTH is one of the most important peptide hormones that regulate calcium and phosphorus homeostasis and bone remodeling [6]. Estrogen can decrease the expression of PTH and lead to a decrease in serum levels of PTH [7]. Estrogen also inhibits PTH-stimulated adenylate cyclase activity and thus PTH-stimulated bone resorption [8]. In addition, estrogen shortage has been found to increase serum levels of BGP (Bone Gla Protein) in postmenopausal women. BGP, also known as γ-carboxyglutamic acid protein, is a hormone-like peptide synthesized and secreted by osteoblasts [9]. BGP is not only an indicator of *in vivo* bone formation rate but also a positive regulator of bone mineralization.

In addition to these hormones, estrogen inhibits bone resorption by suppressing secretion of key osteolytic cytokines such as IL-1 and IL-6 by bone marrow stromal cells and osteoblasts. These cytokines are positive regulators of osteoclast activity and bone resorption [10], [11]. Besides, these cytokines may crosstalk with each other to synergistically promote osteoclastogenesis and bone resorption. For example, IL-1, including both IL-1α and IL-1β subunits, can induce osteoblasts to secrete IL-6 to further promote bone resorption [11]. Estrogen, IL-1β, and IL-6 also regulate PGE production, which promotes proliferation and differentiation of osteoclast precursors. Previous studies have reported that PGE_2_ could also stimulate bone resorption by increasing IL-6 transcription [12], [13]. Furthermore, IL-1 was found to increase COX-2 expression at the mRNA and protein levels, leading to an increase in prostaglandin production. The stabilization of COX-2 mRNA is the most important basis of continued increase in COX-2 protein [14].

Previous studies have shown that increased physical activity is effective in maintaining or even increasing bone and muscle mass in postmenopausal women [15]. Appropriate long-term exercise helps to prevent the development of postmenopausal osteoporosis [16]. Exercise is also effective in maintaining BMD in early postmenopausal women. Kemmler et al. reported that exercises that can improve muscle function might improve overall fitness, maintain bone health, and reduce fall risk [17]. Thus, exercise has been proposed as a preventative measure for osteoporosis. However, the exact mechanisms by which exercise regulates bone remodeling and improves bone quality remain unclear. In animal studies, exercise is generally considered to increase the peak bone mass. Joo et al. showed that treadmill running improves trabecular bone microarchitecture, bone density, and cortical geometry in young growing female rats [18]. Tromp et al. showed that mechanical loading has beneficial effects on bone mineral content (BMC) and bone mineral density of ovariectomized rats [19]. Sakakura et al. showed that exercise has a beneficial effect on tibia and the neck of the condyloid process in ovariectomized rats, probably by increasing serum levels of progesterone [20]. However, how exercise exerts its beneficial effects on bone is not well understood.

We hypothesize that exercise executes its positive influence on bone mass and quality by regulating the synthesis of hormones and cytokines that are involved in bone remodeling. We found that exercise escalates the levels of estrogen in OVX rats, which may suppress the expression of bone resorption stimulators such as CT, IL-1, and IL-6, to inhibit bone resorption. Moreover, we found that exercise also increases bone formation in ovariectomized rats although the mechanisms need further investigation. The results demonstrate that exercise assists in preventing bone loss in rat model of postmenopausal osteoporosis.

## Materials and Methods

### Animals

This study was carried out in strict accordance with the recommendations from the Ethical Committee of Shanghai University of Sports on the Care and Use of Animal Subjects in Research (Permit Number: [2008](2)), following the protocols approved by the committee. All animals were housed in a temperature- and humidity-controlled environment with a 12 h light/12 h dark cycle with free access to food (standard laboratory chow) and water *ad libitum.* All surgery was performed under sodium pentobarbital anesthesia, and all possible efforts were made to minimize suffering.

### Surgical Procedures

A total of 63 female Wistar rats (200 g to 220 g) were obtained from the Laboratory Animal Center of the Academy of Military Medical Sciences, PCLA (License No. SCXK-(military) 2002-001) and maintained in the Laboratory Animal Rooms (License No. SYK11-00-0039) of the Institute of Basic Theory, Chinese Academy of Chinese Medical Sciences. The rats were randomly divided into five groups: control group (n = 13), sham-operated group (n = 13), OVX group (n = 13), DES-OVX group (n = 13), and Ex-OVX group (n = 11). Rats in the OVX group, the DES-OVX group, and the Ex-OVX group were ovariectomized. To remove the ovaries, rats were anesthetized using an intraperitoneal injection of pentobarbital sodium (48 mg/kg) while in prone position. They were shaved along the midaxillary line, 0.5 cm away from the lateral border of the spine below the last rib, exposing the surgical field. An incision on the skin was made on one side through the musculature and peritoneum, and the ovary was subsequently retracted and removed. Tubal ligation was performed, and then the wound was closed. The same procedure was repeated on the other side. The procedure was also performed on rats belonging to the sham-operated group, except that the ovaries of these rats were retracted and put back into their abdomen and some adipose tissues around their ovaries were removed.

### Physical exercise protocol for animals

Two and a half months after surgery, a two-week acclimation period of wheel running training was conducted. Rats in the Ex-OVX group were given access to a running wheel (40 cm diameter, homemade) for 10 min on d 1, with an increase of 10 min/d thereafter until 1 h/d was reached. Wheel revolutions and running distance were counted and automatically recorded using a counter. Three months after surgery, rats in the Ex-OVX group were made to run 200 revolutions (251.2 meters) in the running wheel every day, 6 d/wk for 3 months. Rats belonging to the DES-OVX group were intragastrically administered with Diethylstilbestrol (0.045 mg/kg; concentration, 0.0045 mg/ml; Beijing Yimin Pharmaceutical Co., Ltd., Batch No. H11020899). The control, OVX, and sham-operated groups were given distilled water every day, 6 d/wk for 3 months.

### Animal tissue preparation

Rats in all groups were given two intraperitoneal injections of tetracycline hydrochloride (30 mg/kg; Aozehui Technology and Trade Company, US22105) 3 and 16 d before exercising. Five and a half months after surgery, all rats were anesthetized using an intraperitoneal injection of pentobarbital sodium (45 mg/kg; Beijing Tongxian Yucai Fine Chemical Works, Batch No. 950427). When rats appeared to clamping as judged by independent animal care personnel with no knowledge of the protocol design, they were euthanized by Carbon monoxide in a box. Then rats’ blood samples were collected from the femoral arteries. The tibias were harvested, with the right tibias being used for undecalcified bone sections to determine bone morphometric indices and the left ones being used for decalcified bone frozen sections for in situ hybridization to detect IL-1β, IL-6, and COX-2 mRNA levels. The blood obtained from the femoral arteries was centrifuged (2000 r/min) and the supernatant was collected. The serum was transferred to an Eppendorf tube and preserved at −20°C for future detection of E2, calcitonin, osteocalcin, and parathyroid hormone.

### Bone histomorphometry

The proximal one-third of the right tibias of the rats were obtained and the soft tissues, along with some cortex at both ends, were removed. The samples were dehydrated in increasing concentrations of ethanol (twice at each concentration, each time for 24 h), and xylene was used as a transparency agent (50% ethanol + 50% xylene 30 min, and xylene 30 min twice). The next step was resination of the samples by immersing them in the solutions described below: Solution I: 75 ml methyl methacrylate (Beijing Yili Fine Chemical Co. Ltd., Batch No. 960523), 25 ml dibutyl phthalate (DBP; Beijing Chemical Reagent Company, Batch No. 970628); Solution II: 1 g benzoyl peroxide (Beijing Jinlong Chemical Reagent Co. Ltd., Batch No. 20000420) added to Solution I; Solution III: 2.5 g benzoyl peroxide added to Solution I. Each of these solutions was mixed using a magnetic stirrer. The specimens were sequentially immersed in Solutions I, II, and III, for 36 hrs each. Afterwards, 5 ml of Solution III was injected into a small bottle containing penicillin. The specimens were placed in the bottle. The closed bottle was then placed in an incubator. Polymerization was performed at 40°C for 3 d to 4 d. A hard, colorless, transparent block with embedded samples was formed. The block was taken out and shaped using a file. The resulting undecalcified bone sections were cut at a thickness of 5 or 10 µm on a microtome (Autocut 2040, Reichert-Jung, Heidelberg, Germany) with a tungsten steel blade. The 5 µm-thick undecalcified bone sections were placed in methyl methacrylate for 2 h and then twice in xylene, each time for 1 h. After deresination, the sections were again dehydrated with increasing concentrations of ethanol and stained with toluidine blue. The 10 µm-thick undecalcified bone sections were directly subjected to fluorescence observation.

Leica QWin image analysis software was used to determine the morphometric indices of the undecalcified bone sections using the method reported by Liu [21]. The morphometric indices of trabecular bone, including trabecular bone volume (TBV%), total resorption surface (TRS%), trabecular formation surface (TFS%), and mineralization rate (MAR), as well as those of the inner surface of the cortex bone, including osteoid seam width (OSW) and bone cortex mineralization rate (mAR), were calculated.

### Quantitation of IL-1β, IL-6, and COX-2

The proximal one-third of the left tibias of the rats was harvested with the soft tissues removed. The tibias were then fixed in 4% paraformaldehyde for 24 h, fully rinsed using PBS, and decalcified for 3 wk at 4°C using 10% EDTA/PBS. The decalcification solution was changed every other day. The decalcified bones were rinsed with PBS and then dehydrated in 15% sucrose solution/PBS for 24 h. Frozen sections were later cut at 10 µm on a refrigerated microtome (BRIGHT–5030, UK).

Immunohistochemical staining was used to detect IL-1β, IL-6, and COX-2 protein levels. IL-1β, IL-6, and COX-2 mRNA levels in the rat tibias were determined using in situ hybridization kits (Wuhan Boster Biological Technology, Ltd.). Oligonucleotide probes for IL-1β, IL-6, and COX-2 were labeled with digoxin. The mRNA sequences transcribed from the human IL-1β genes are shown in **Table 1**. The only difference among the sequences of rat, mouse, and human lies on 1 bp/90 bp. The mRNA sequences transcribed from the rat and mouse IL-6 genes are shown in **Table 2**. The mRNA sequences transcribed from the human COX-2 target genes are shown in **Table 3**. The only difference among the sequences of rat, mouse, and human lies on 5 bp/90 bp. In the morphometric analysis that used the Leica Q Win image analysis software, a positive reaction was indicated by brown coloration of the cytoplasm. More than five high-power fields (×400) below the epiphyseal plate within the medullary cavity were randomly picked and analyzed. The mRNA and protein level of IL-6 and COX-2 in osteoblasts was determined by the ratio of positive cells area per trabecular area in high-power field, and the mRNA and protein level of IL-1β in bone marrow was determined by the ratio of positive cells area per high-power field (without trabecular area), the mean was calculated and recorded as the percentage of positively reacting cells (%).

**Table 1:** The mRNA sequence of IL-1β target genes.

Group	The mRNA sequence of IL-1β target genes							
No.1	5′	ACAAA	ATACC	TGTGG	CCTTG	GGCCT	CAAGG	3′
No.2	5′	GAAGA	AGATG	GAAAA	GCGAT	TTGTC	TTCAA	3′
No.3	5′	CAACT	GGTAC	ATCAG	CACCT	CTCAA	GCAGA	3′

**Table 2:** The mRNA sequence of IL-6 target genes.

Group	The mRNA sequence of IL-6 target genes							
No.1	5′	CTCCG	CAAGA	GACTT	CCAGC	CAGCT	GCCTT	3′
No.2	5′	CTTCC	AAACT	GGATA	TAACC	AGGAA	ATTTG	3′
No.3	5′	ATTTC	TAAAG	GTCAC	TATGA	GGTCT	ACTCG	3′

**Table 3:** The mRNA sequence of COX-2 target genes.

Group	The mRNA sequence of COX-2 target genes							
No.1	5′	ATGTA	TGAGT	GTGGG	ATTTG	ACCAG	TATAA	3′
No.2	5′	GAACG	TTGTG	AATAA	CATTC	CCTTC	CTTCG	3′
No.3	5′	TGCCT	CAATT	CAGTC	TCTCA	TCTGC	AATAA	3′

### Measurement of serum CT, BGP, PTH, and E_2_

ELISA assays were adapted to determine the levels of serum CT, BGP, PTH, and E_2_. ELISA kits for rat BGP, CT, and PTH were purchased from Market Company (Batch Nos. RE030, IR108, and IR067) and the E_2_ kit was purchased from Tianjing Jiuding Biomedical Engineering Ltd. (Batch No. ESBL4287).

### Statistical analysis

Data are presented as mean ± standard deviation. Statistical evaluation was performed using one-way ANOVA. Data were compared between groups, and a Q-test was performed. Significant differences were considered at *p<0.05* and highly significant differences at *p<0.01*.

## Results

In this study, we studied the effects of exercise on bone remodeling parameters of ovariectomized rats by histomorphometry and explored whether bone remodeling-related hormones and cytokines are involved.

### Effect of exercise on bone histomorphometric parameters

**Figure 1:**
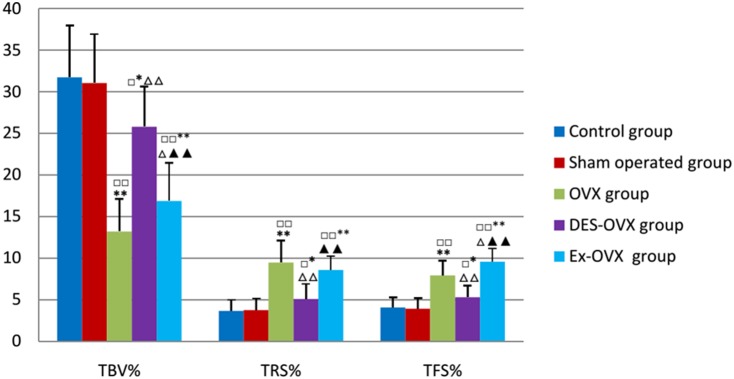
TBV, TRS, and TFS percentages in the five groups. compared with the control group: ^□^ *p*<0.05, ^□□^ *p*<0.01; compared with the sham-operated: *p<0.05, **p<0.01; compared with the OVX group: ^△^ *p*<0.05, ^△△^ *p*<0.01; compared with the DES-OVX group: ^▴▴^ *p*<0.01.

**Figure 2:**
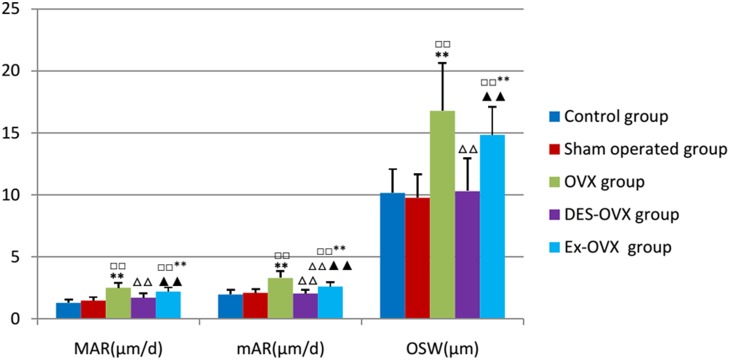
MAR, mAR, and OSW in the five groups. compared with the control group: ^□□^ *p*<0.01; compared with the sham-operated: **p<0.01; compared with the OVX group: ^△△^ *p*<0.01; compared with the DES-OVX group: ^▴▴^ *p*<0.01.

As shown in **Table 4** **and** **Figure 1**, the TBV% of the OVX group was significantly lower than the control and the sham-operated groups, which was accompanied by increased TRS, confirming that ovariectomy led to increased bone resorption and bone loss in rats. Moreover, the OVX group rats also showed higher levels of TFS%, MAR, mAR, and OSW than the control and sham-operated group rats (**Table 5** **and** **Figure 2**), suggesting that these rats also displayed increased bone formation, which is likely caused by the coupling between bone resorption and formation. Thus, ovariectomized rats showed high bone turnover rate, with the increase in bone resorption being greater than that in bone formation. These findings are in accordance with previous studies and suggest that the surgery was successful in this study. To confirm these findings, we also treated ovariectomized rats with DES and found that DES almost completely reversed the bone loss and restored the bone parameters. In addition, none of the parameters was significantly different between the sham-operated group and the normal control group, which excludes the impact of surgical factors.

**Table 4:** TBV, TRS, and TFS percentages in the five groups.

Group	TBV%	TRS%	TFS%
control group	31.75±6.23	3.65±1.32	4.07±1.21
sham-operated group	31.05±5.88	3.76±1.37	3.91±1.29
OVX group	13.21±3.91^□□**^	9.48±2.63^□□**^	7.92±1.76^□□**^
DES-OVX group	25.81±4.79^□*△△^	5.09±1.79^□*△△^	5.31±1.37^□*△△^
Ex-OVX group	16.90±4.52^□□**△▴▴^	8.59±1.65^□□**▴▴^	9.58±1.56^□□**△▴▴^

compared with the control group: ^□^ *p*<0.05, ^□□^ *p*<0.01;

compared with the sham-operated: ^*^ *p*<0.05, ^**^ *p*<0.01;

compared with the OVX group: ^△^ *p*<0.05, ^△△^ *p*<0.01;

compared with the DES-OVX group: ^▴▴^ *p*<0.01.

**Table 5:** The levels of MAR, mAR, and OSW in the five groups.

Group	MAR (µm/d)	mAR (µm/d)	OSW (µm)
control group	1.31±0.25	1.98±0.38	10.17±1.90
sham-operated group	1.49±0.27	2.11±0.32	9.77±1.90
OVX group	2.52±0.40^□□**^	3.31±0.56^□□**^	16.79±3.85^□□**^
DES-OVX group	1.73±0.33^△△^	2.06±0.31^△△^	10.32±2.63^△△^
Ex-OVX group	2.22±0.33^□□**▴▴^	2.62±0.36^□□**△△▴▴^	14.85±2.25^□□**▴▴^

compared with the control group: ^□□^ *p*<0.01;

compared with the sham-operated: ^**^ *p*<0.01;

compared with the OVX group: ^△△^ *p*<0.01;

compared with the DES-OVX group: ^▴▴^ *p*<0.01.

More importantly, exercise significantly increased the bone mass in the Ex-OVX group rats, which could be attributable to a decrease in bone resorption and an increase in bone formation, as TRS% was decreased whereas TFS% was increased compared to the OVX group. These bone histomorphometric parameters were measured in trabecular bones. On the other hand, the cortical bone parameters including MAR, mAR, and OSW were slightly decreased in the EX-OVX rats compared to OVX rats. These results suggest exercise can inhibit bone resorption and increase trabecular bone formation, which helps to prevent further bone loss in ovariectomized rats.

### Effect of exercise on serum levels of CT, BGP, PTH, and E_2_

**Figure 3:**
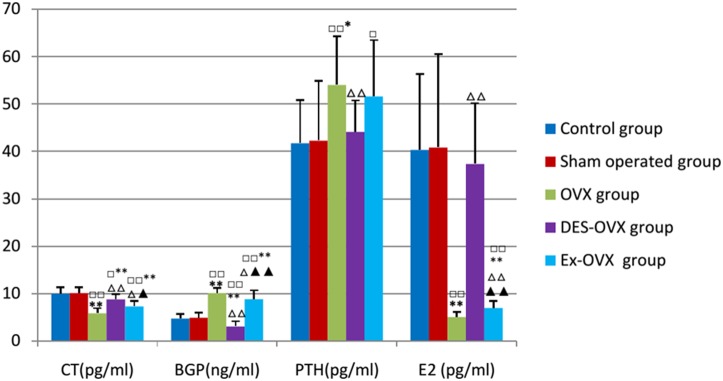
CT, BGP, PTH, and E_2_ in the five groups. compared with the control group: ^□^ *p*<0.05, ^□□^ *p*<0.01; compared with the sham-operated: *p<0.05, **p<0.01; compared with the OVX group: ^△^ *p*<0.05, ^△△^ *p*<0.01; compared with the DES-OVX group: ^▴^ *p*<0.05, ^▴▴^ *p*<0.01.

We then analyzed some of the hormones that are involved in bone remodeling, especially the hormones whose levels are altered in post-menopausal osteoporotic patients and animal models. As shown in **Table 6** **and** **Figure 3**, rats in the OVX group had much lower E_2_ whereas DES-OVX rats showed E2 levels that were comparable to the control rats. Interestingly, exercise significantly increased serum levels of E2, which might be synthesized and secreted by organs other than the ovary [22]. The increase in E2 levels was expected to inhibit bone resorption.

**Table 6:** The levels of CT, BGP, PTH, and E_2_ in the five groups.

Group	CT (pg/ml)	BGP (ng/ml)	PTH (pg/ml)	E2 (pg/ml)
control group	9.98±1.33	4.76±0.90	41.72±9.14	40.36±15.91
sham-operated group	10.13±1.24	4.94±1.03	42.30±12.52	40.87±19.61
OVX group	5.88±1.02^□□**^	10.14±1.06^□□**^	54.03±10.27^□□*^	5.09±1.07^□□**^
DES-OVX group	8.78±1.09^□**△△^	3.09±1.06^□□**△△^	44.13±6.60^△△^	37.38±12.78^△△^
Ex-OVX group	7.30±1.61^□□**△▴^	8.85±1.81^□□**△▴▴^	51.65±11.83^□^	6.98±1.46^□□**△△▴▴^

compared with the control group: ^□^ *p*<0.05, ^□□^ *p*<0.01;

compared with the sham-operated: ^*^ *p*<0.05, ^**^ *p*<0.01;

compared with the OVX group: ^△^ *p*<0.05, ^△△^ *p*<0.01;

compared with the DES-OVX group: ^▴^ *p*<0.05, ^▴▴^ *p*<0.01.

Moreover, the levels of CT, a negative regulator of osteoclast differentiation and activity, were decreased in OVX rats. On the other hand, the levels of PTH, a positive regulator of bone resorption, were increased in the OVX group compared to the control and the sham-operated groups. Additionally, the change in these two hormones was restored to close to normal levels in the DES-OVX group. These results suggest that estrogen deficiency led to a decrease in CT but an increase in PTH in ovariectomized rat. Furthermore, the levels of BGP, an osteoblast-produced hormone that promotes bone mineralization and acts as an indicator of in vivo bone formation rate, was also increased in ovariectomized rats. These results are consistent with our finding that estrogen deficiency leads to enhanced bone formation, in which BGP might play a positive role.

We also found that rats in the Ex-OVX group showed higher levels of CT and lower levels of BGP than the OVX rats, without significantly changing the levels of PTH. Note that the Ex-OVX group still showed lower levels of CT and E2, as well as higher levels of BGP and PTH, than the control and the sham-operated groups. These findings suggest that exercise may inhibit bone loss and prevent osteoporosis by regulating the serum levels of CT, PTH, and BGP.

### Alteration of protein levels of IL-1β in the bone marrow and IL-6 and COX-2 in tibias

**Figure 4:**
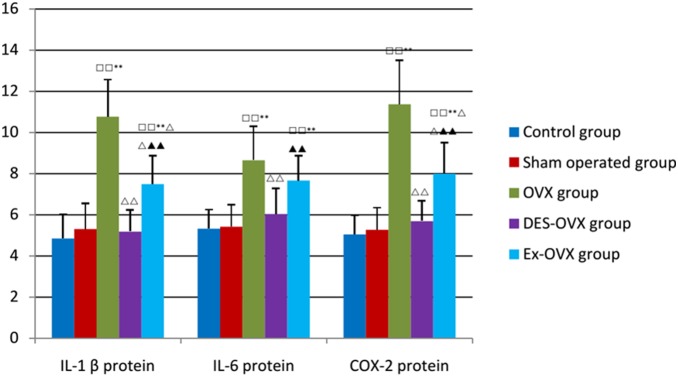
IL-1β, IL-6, and COX-2 protein expression in the five groups. compared with the control group: ^□□^ *p*<0.01; compared with the sham-operated: **p<0.01; compared with the OVX group: ^△△^ *p*<0.01; compared with the DES-OVX group: ^▴▴^ *p*<0.01.

As shown in **Table 7** **and** **Figure 4**, the OVX rats showed an increase in the protein levels of IL-1β, IL-6, and COX-2 compared to the control and the sham-operated groups, which were all restored to normal levels in the DES-OVX group, suggesting that estrogen deficiency is the reason behind the alteration of the expression of these proteins. Moreover, we found that the Ex-OVX group had much lower levels of IL-1β, IL-6, and COX-2 than the OVX group. These results suggest that exercise could partially inhibit the expression of IL-1β, IL-6, and COX-2, which helps to suppress bone resorption.

**Table 7:** IL-1β, IL-6, and COX-2 protein levels in the five groups.

Group	IL-1βprotein expression (%)	IL-6protein expression (%)	COX-2protein expression (%)
control group	4.84±1.17	5.32±0.92	5.04±0.94
sham-operated group	5.30±1.24	5.41±1.07	5.26±1.08
OVX group	10.76±1.80^□□**^	8.64±1.65^□□**^	11.36±2.14^□□**^
DES-OVX group	5.18±1.05^△△^	6.03±1.25^△△^	5.69±0.99^△△^
Ex-OVX group	7.48±1.37^□□**△△▴▴^	7.65±1.20^□□**▴▴^	7.99±1.50^□□**△△▴▴^

compared with the control group: ^□□^ *p*<0.01;

compared with the sham-operated: **p<0.01;

compared with the OVX group: ^△△^ *p*<0.01;

compared with the DES-OVX group: ^▴▴^ *p*<0.01.

### Alteration of mRNA levels of IL-1β in the bone marrow and IL-6 and COX-2 in tibias

**Figure 5:**
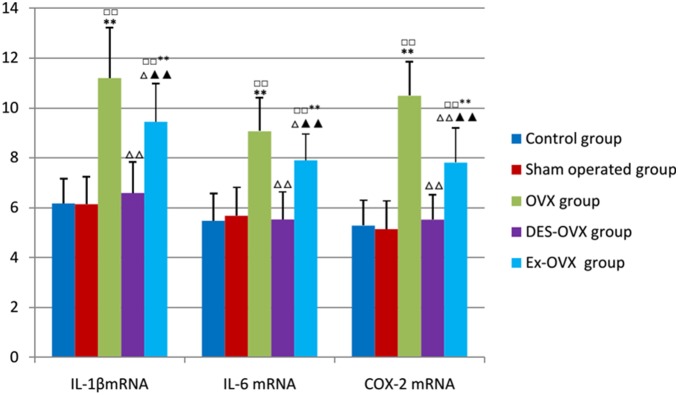
IL-1β, IL-6, and COX-2 mRNA expression in the five groups. compared with the control group: ^□□^ *p*<0.01; compared with the sham-operated: **p<0.01; compared with the OVX group: ^△^ *p*<0.05, ^△△^ *p*<0.01; compared with the DES-OVX group: ^▴▴^ *p*<0.01.

To validate the above finding, we also analyzed the mRNA levels of these proteins on bone sections by in situ hybridization. As shown in **Table 8** **and** **Figure 5**, the OVX rats showed much higher levels of IL-1β, IL-6, and COX-2 mRNA than the control and the sham-operated groups, which were restored to normal levels in the DES-OVX group. Moreover, the Ex-OVX group rats showed much lower levels of IL-1β, IL-6, and COX-2 mRNA than the OVX group. These results suggest that exercise could inhibit the expression of these cytokines at the mRNA level.

**Table 8:** IL-1β, IL-6, and COX-2 mRNA levels in the five groups.

Group	IL-1βmRNA expression (%)	IL-6mRNA expression (%)	COX-2mRNA expression (%)
control group	6.17±0.99	5.48±1.08	5.28±1.01
sham-operated group	6.14±1.10	5.68±1.13	5.14±1.13
OVX group	11.20±2.02^□□**^	9.07±1.33^□□**^	10.49±1.36^□□**^
DES-OVX group	6.59±1.23^△△^	5.53±1.10^△△^	5.52±1.00^△△^
Ex-OVX group	9.45±1.53^□□**△▴▴^	7.90±1.05^□□**△▴▴^	7.81±1.38^□□**△△▴▴^

compared with the control group: ^□□^ *p*<0.01;

compared with the sham-operated: ^**^ *p*<0.01;

compared with the OVX group: ^△^ *p*<0.05, ^△△^ *p*<0.01;

compared with the DES-OVX group: ^▴▴^ *p*<0.01.

## Discussion

Ovariectomized rats and mice are well-established models for the study of the pathogenesis of postmenopausal osteoporosis and are widely used for evaluation and development of new drugs for treatment of postmenopausal osteoporosis [23]–[25]. Here we used the rat model to study the possible effect of exercise on bone remodeling. Consistent with the previous results, we found that ovariectomized rats exhibited a significant decrease in bone mass, whereas bone resorption indexes TRS%, PTH, and bone formation markers TFS%, MAR, OSW, mAR, and BGP, were all significantly increased. This finding indicates that ovariectomy increases bone turnover rate, wherein bone resorption outpaces bone formation, leading to the development of osteoporosis. Moreover, DES was found to reverse all these changes in ovariectomized rats.

Previous studies have shown that osteoporosis-related bone loss in postmenopausal rat was partially prevented by moderate exercise [26]–[28]. Our findings support this concept and provide more histomorphometric evidence. Rats in the EX-OVX group showed higher TBV% than those in the OVX group, indicating that running wheel improves cancellous bone in ovariectomized rats. Compared to the OVX group, the EX-OVX group rats showed a modest decrease in bone resorption rate, which is correlated with an increase in serum levels of estrogen and CT. On the other hand, exercise increased bone formation, especially of trabecular bones, which is reflected by an increase in TFS%. However, bone formation rate at the endosteal surface of cortical bones in the EX-OVX group was lower than that in the OVX group, suggesting that exercise may differentially regulate bone formation in trabecular and cortical bones. It seems that exercise can partially prevent estrogen deficiency-induced bone loss by suppressing bone resorption and increasing bone formation. While it is still debated whether decreased bone resorption or increased bone formation is the main reason behind exercise-induced bone mass elevation [29], [30], our results support the involvement of both events.

Estrogen exerts its anti-osteoporotic activity by directly or indirectly regulating bone resorption [31], [32]. While there is convincing evidence that estrogen, acting via estrogen receptor α, stimulates osteoclast apoptosis and suppresses osteoblasts apoptosis [33], estrogen has been shown to act on MSCs and osteoblasts to regulate the expression of cytokines and growth factors that control osteoclast differentiation and activity. Since long-term HRT has adverse effects, caution is expected in choosing HRT for prevention and treatment of osteoporosis in women [34], [35]. On the other hand, a number of studies have shown that appropriate intensities of exercise can increase serum levels of estradiol and testosterone, which helps to improve osteoblast proliferation and activity, leading to an increase in bone mass and density [36], [37]. However, some clinical studies showed that the estrogen levels were unchanged in response to exercise [38]. This discrepancy could be caused by the difference in the intensities of exercise, the age and ethnicity of the patients, or both. Nevertheless, this study shows that wheel running increases serum E_2_ in ovariectomized rats, which can slow down bone loss induced by ovariectomy. In addition, the levels of CT, an inhibitor of osteoclast differentiation and activity [5], are also decreased after ovariectomy, likely due to estrogen shortage. Indeed, previous studies have shown that estrogen stimulates the synthesis of CT [4]. We found that exercise also increases the levels of CT in ovariectomized rats, which might be a result of increased estrogen.

We found that the OVX group rats showed higher levels of BGP and PTH than the control group. BGP is a hormone-like peptide synthesized and secreted by osteoblasts and is significantly increased in the serum of postmenopausal women [9]. It has pro-minerilization function and is an indicator of in vivo bone formation rate. Thus, it is possible that estrogen regulates bone formation and bone resorption thorugh these two hormones as well. The observation that supplementation with diethylstilbestrol reversed the alteration in BGP and PTH expression in ovariectomized rats suggests that estrogen deficiency is the underlying cause for the change in BGP and PTH levels. In addition, we found that exercise decreases the levels of BGP in ovariectomized rats, which might be due to the increase in the E2 levels.

Our study further showed that the protein and mRNA levels of the bone marrow IL-1β and osteoblastic IL-6 and COX-2 were increased in ovariectomized rats. The change in the levels of these proteins was highly related to E_2,_ as there is no significant difference between the DES-OVX group and the sham-operated group. Charatcharoenwitthaya et al. demonstrated that in humans, like in rodents, part of the effects of estrogen deficiency on increased bone resorption is mediated by IL-1 [39]. Moreover, IL-6 can enhance osteoclastogenesis in vivo and in vitro, which could be prevented by 17 beta-estradiol or an antibody against IL-6. Thus, IL-1 and IL-6 play critical roles in estrogen-regulated osteoclastogenesis. Moreover, IL-1 and IL-6 regulate the expression of Cox-2 and therefore the production of PGE_2_, which also increases osteoclast activity and bone resorption [40]. Our study revealed that exercise could effectively decrease the mRNA and protein levels of IL-1, IL-6, and Cox-2 in ovariectomized rats. These results, taken together, suggest that exercise may regulate serum levels of the hormones and cytokines via E2, to inhibit bone resorption and to prevent further bone loss in estrogen deficiency-induced osteoporosis models.

While therapeutic HRT inhibits both osteogenesis and bone resorption, with inhibition of bone resorption being the dominant effect, we found that exercise inhibits bone resorption but increases osteogenesis. The dual beneficial effects of exercise may have positive impact on bone geometry in addition to bone mass and density. As such, an increasing number of researchers are considering using exercise or combination of exercise with drugs such as bisphosphonates to treat osteoporosis [41], [42].

## Conclusions

Overall, our study revealed that exercise acts on both bone formation and bone resorption to slow down bone loss in ovariectomized rat models. Exercise not only increases the serum levels of E2 but also affects the expression of hormones that play critical roles in bone remodeling, such as CT, BGP and PTH. Moreover, exercise also inhibits the expression of IL-1, IL-6, and Cox-2 to negatively regulate osteoclastogenesis and bone resorption. However, the interaction between these cytokines and hormones needs further investigation.

## Supporting Information

Checklist S1 **The ARRIVE Guidelines Checklist: Animal Research:Reporting** ***In Vivo*** **Experiments.**
(DOC)
